# Population Pharmacokinetics, Exposure-Response, and Probability of Target Attainment Analyses for Tedizolid in Adolescent Patients with Acute Bacterial Skin and Skin Structure Infections

**DOI:** 10.1128/AAC.00895-21

**Published:** 2021-11-17

**Authors:** Dan Li, Philip E. Sabato, Benjamin Guiastrennec, Aziz Ouerdani, Hwa-Ping Feng, Vincent Duval, Carisa S. De Anda, Pamela S. Sears, Margaret Z. Chou, Catherine Hardalo, Natalya Broyde, Matthew L. Rizk

**Affiliations:** a Merck & Co., Inc., Kenilworth, New Jersey, USA; b Certara, Data Science Services, Basel, Switzerland; c Certara, Data Science Services, Paris, France

**Keywords:** pediatric, pharmacodynamics, pharmacokinetics, skin and soft tissue infections, tedizolid

## Abstract

Tedizolid phosphate is an oxazolidinone antibacterial agent approved for the treatment of Gram-positive acute bacterial skin and skin structure infections (ABSSSIs) in patients aged ≥12 years. To support the use of tedizolid phosphate in adolescents with ABSSSIs, a population pharmacokinetic (PK) model, developed using adult and pediatric data, was updated to include PK data from a phase 3 clinical trial (PN012) that evaluated the safety and efficacy of once-daily oral or intravenous 200-mg tedizolid phosphate treatment in adolescents (12 to <18 years) with ABSSSIs, along with emerging data from a phase 1 trial (PN013) in children (2 to <12 years). Updated PK parameter estimates remained similar to those of the previous model. Body weight was a statistically significant covariate on clearance and volume parameters, with no clinically meaningful effects on exposure in adolescents. Tedizolid exposures in adolescents from PN012 were slightly higher with largely overlapped area under the concentration-time curve distribution compared with adults from previous phase 2 and 3 trials. The probability of PK/pharmacodynamic target attainment at the MIC susceptibility breakpoint of 0.5 μg/ml for Staphylococcus and Streptococcus sp. was 100%. As most participants from the PN012 trial were cured, no significant exposure-efficacy relationship was identified. Tedizolid exposures were similar between participants with and without a safety event from PN012; no clear relationship was detected between exposure and safety. Despite lower body weight and higher exposures in adolescents, safety profiles in adolescents were similar those in adults. These results support the 200-mg, once-daily intravenous or oral dose of tedizolid phosphate in adolescents with ABSSSIs.

## INTRODUCTION

Tedizolid phosphate is an oxazolidinone prodrug antibacterial agent developed for oral or intravenous (i.v.) administration in the management of Gram-positive infections, including those caused by methicillin-resistant or -susceptible Staphylococcus aureus in adult and pediatric patients aged ≥12 years (1–6). Tedizolid phosphate administered as an oral or i.v. dose was approved initially in adults for the treatment of Gram-positive acute bacterial skin and skin structure infections (ABSSSIs) based on two pivotal randomized, double-blind, multicenter, controlled, phase 3 clinical trials (ESTABLISH-1 and ESTABLISH-2) (7, 8). These trials demonstrated that oral or i.v. dosing of tedizolid phosphate, administered as a 200-mg once-daily dose for 6 days, was noninferior to a 600-mg dose of linezolid, administered twice daily for 10 days, based on early clinical response at 48 and 72 h. In addition, tedizolid phosphate demonstrated an overall favorable safety profile compared with linezolid and had a lower incidence of gastrointestinal adverse events.

A phase 1 pharmacokinetic (PK) trial assessed tedizolid phosphate in adolescents 12 to <18 years of age who were receiving prophylaxis or treatment for confirmed or suspected Gram-positive infections (9). Results of the PK analysis showed that the adult dose of 200 mg of tedizolid phosphate was appropriate for further evaluation in this adolescent group. Therefore, the efficacy and safety of 200 mg of tedizolid phosphate were assessed in adolescents with ABSSSIs in a single-blind comparative phase 3 trial (10). The efficacy of tedizolid phosphate was similar to that of standard-of-care comparators (vancomycin, linezolid, clindamycin, cefazolin, cephalexin, and flucloxacillin), and the safety profile was favorable (10).

Tedizolid phosphate is a prodrug that is hydrolyzed by nonspecific phosphatases to tedizolid, the microbiologically active form (11, 12). The PK of tedizolid has been studied extensively. In adults, the absolute bioavailability of tedizolid is >80%, the time to maximum concentration is achieved within approximately 3 hours of oral dosing, and steady-state plasma concentrations are reached within 3 days of the initial daily dose (13–15). Tedizolid is moderately protein bound in human plasma (∼80%), and microdialysis studies have shown that the drug is well distributed into skin and soft tissue where unbound tedizolid concentrations are roughly equivalent to the free concentrations in plasma (15). These data indicate that tedizolid plasma concentrations can serve as an appropriate surrogate for tissue concentration (15). Previous reports describing the pharmacodynamics (PD) of tedizolid in neutropenic mouse methicillin-resistant and methicillin-susceptible S. aureus infection models were leveraged to determine the PK/PD target, and a correction factor was applied to account for higher lung penetrations in humans compared with those in mice (16–18). Area under the concentration-time curve for the free, unbound fraction of a drug (*f*AUC)/MIC ratio of ≥3 was the PK/PD target that best described variability in the PD response against methicillin-resistant and -susceptible S. aureus in preclinical dose-fractionation studies conducted in a murine model of ABSSSI (18, 19). This ratio was adequate to achieve stasis in immunocompetent mice (20).

A population PK (popPK) model for tedizolid in adults was developed previously using plasma concentration data from participants in several phase 1 trials, a phase 2 trial of complicated skin and skin structure infections, and two phase 3 trials conducted in adults and pediatric participants with ABSSSIs (21). Here, we report on the development of an updated tedizolid popPK model and a related analysis, including an estimation of probability of target attainment (PTA) with tedizolid therapy and exposure-efficacy and exposure-safety analyses in adolescent participants.

## RESULTS

### Participants.

A summary of participant baseline characteristics stratified by trial is shown in Tables A1 and A2 in the supplemental material. Across the 16 trials that contributed data for this analysis, 50% to 100% of participants in each trial were White. Data from 1,312 trial participants contributed to this analysis, which included 945 adults with ABSSSIs (72.0%), 223 healthy participants (17.0%), 41 hospitalized participants (3.1%), and 103 adolescents with ABSSSIs (7.9%; 91 participants from PN012 and 12 participants from trials 104, 112, 113, and 16121). Among the 132 (10.1%) participants who were <18 years of age, 20 (1.5%) were adolescents hospitalized with suspected Gram-positive infections that were not confirmed as ABSSSIs.

Overall, 9,793 tedizolid plasma PK samples were available for analysis after excluding those with postdose measurements that were below the limit of quantitation (23 samples [0.2%]) and samples with no measurable tedizolid concentration (i.e., missing; 78 samples [0.8%]). Among the total samples analyzed, 8,942 (91.3%) were from the adult population and 851 (8.7%) were obtained from participants aged ≤18 years. After the exclusion of all outliers (37 samples [0.4%] from 27 participants, including 8 participants from PN012), 9,756 PK observations were used to reestimate the popPK model parameters. Covariates (body weight, ABSSSI, and diabetes) for participants with excluded data points were within the normal range of the population (data not shown). Among adolescent participants, body weight was within the normal range, and diabetes was not reported (data not shown). Goodness-of-fit plots for each individual study showed that the model was adequate to describe the data and suggested there was a good agreement between the observed and model-predicted tedizolid concentrations in both adolescent and adult participants (see Fig. A1 in the supplemental material).

### PopPK analysis.

The updated popPK model used a two-compartment model with linear elimination and sigmoidal absorption to describe tedizolid PK. Parameter estimates for the updated popPK model are presented in Table 1. Fixed-effects parameters were generally estimated with high precision (relative standard error [RSE], <30%), except for the infection effect on clearance and volume of the central compartment, which were estimated with RSEs of 39.3% and 37.4%, respectively. All random effects parameters were estimated with high precision (RSE, <9%). The effect of body weight on tedizolid PK parameters in participants with ABSSSIs was assessed in the popPK analysis containing both adult and adolescent data. In the final popPK model for tedizolid, body weight was a significant covariate on clearance, intercompartmental clearance, and the volume of distribution in the central and peripheral compartments. ABSSSI was a covariate on clearance and volume in the central compartment, and diabetes was a covariate on volume in the central compartment. Total body weight, ABSSSIs, and diabetes were the only covariate effects present in the model. No clear trends or specific impact of age or creatinine clearance on exposure were predicted. Equations for the main model parameters are provided in Table 1.

**TABLE 1:** PopPK^*a*^ parameter estimates for the final model

Parameter^*b*^	Values for^*c*^:			
	Final model^*d*^		Bootstrap^*e*^	
	Fixed effect (%RSE^*f*^)	IIV, %CV (%RSE^*f*^)	Fixed effect (95% CI)	IIV %CV (95% CI)
Infusion time, fixed (h)	0.810 FIXED	8.26 FIXED	0.810 FIXED	8.26 FIXED
F1	0.857 (0.965)		0.85 (0.834, 0.86)	
Zero-order duration (h)	0.175 (28.3)	258 (7.39)	0.475 (0.441, 0.586)	210 (179, 221)
*K_a_* (h^–1^)	1.47 (9.25)	77.0 (8.36)	0.964 (0.923, 1.03)	74.1 (70.1, 78.4)
Lag time (h)	0.226 (0.0376)	100 (5.47)	0.178 (0.135, 0.227)	119 (101, 129)
CL (liter/h)	5.39 (6.98)	32.2 (2.87)	5.48 (5.34, 5.6)	32.2 (30.4, 34.2)
wt (power model)^*g*^	0.408 (10.2)		0.451 (0.411, 0.492)	
Infection (%) (linear model)	22.0 (39.3)		21.4 (18.7, 24.1)	
*V*_c_ (liter)	58.5 (3.36)	25.2 (4.67)	59.1 (57.9, 60.4)	25.3 (22.6, 28.5)
wt (power model)^*g*^	0.903 (3.53)		0.937 (0.886, 0.975)	
Infection (%) (linear model)	9.87 (37.4)		10.7 (8.81, 13.9)	
Diabetes (%) (linear model)	−14.3 (22.2)		−14.0 (–18.6, −9.49)	
Q (L/h)	1.43 (4.09)		1.44 (1.36, 1.52)	
wt (power model)^*g*^	Same as for CL		Same as for CL	
*V*_p_ (L)	15.6 (2.41)	15.8 (8.54)	15.8 (15.3, 16.4)	16.2 (13.7, 18.5)
wt (power model)^*g*^	0.678 (6.87)		0.677 (0.609, 0.731)	
Correlation CL-*V*_c_ (%)		62.1 (5.28)		61.9 (52.1, 68.5)
RV				
RV for non-phase 3 trials (%)	12.3 (1.36)		12.4 (11.8, 12.9)	
RV for study 104 and phase 3 trials (fold)^*h*^	4.92 (4.63)		4.57 (4.13, 5.34)	
RV for oral data (fold)^*h*^	2.01 (5.22)		2.23 (1.87, 2.66)	

a popPK, population pharmacokinetics.

b Equations for the main model parameters: $CL_{i}=TV_{\mathrm{CL}}\cdot {\left(\frac{\mathrm{WT}}{77.3}\right)}^{\theta_{\mathrm{WT}-\mathrm{CL}}}\cdot \left(1+\theta_{\mathrm{Infec}-\mathrm{CL}}\cdot \mathrm{INFEC}\right)\cdot e^{\eta_{CL_{i}}}$
$Vc_{i}=TV_{\mathrm{Vc}}\cdot {\left(\frac{\mathrm{WT}}{77.3}\right)}^{\theta_{\mathrm{WT}-\mathrm{Vc}}}\cdot \left(1+\theta_{\mathrm{Infec}-\mathrm{Vc}}\cdot \mathrm{INFEC}\right)\cdot \left(1+\theta_{\mathrm{Diab}-\mathrm{Vc}}\cdot \mathrm{DIAB}\right)\cdot e^{\eta_{Vc_{i}}}$ $Vp_{i}=TV_{\mathrm{Vp}}\cdot {\left(\frac{\mathrm{WT}}{77.3}\right)}^{\theta_{\mathrm{WT}-\mathrm{Vp}}}\cdot e^{\eta_{Vp_{i}}}$ where *CL_i_*, *V*_ci_, *V*_pi_, *TV_CL_*, *TV_Vc_*, *TV_Vp_*, and *TV_Q_* represent the individual and typical parameter values, respectively, for CL, *V*_c_, and *V*_p_. θ_WT–xx_ represents the effect of body weight (WT) on the different parameters. *θ_Infec_*_–xx_ represents the relative effect of bacterial infection. *θ_Diab_*_–xx_ represents the relative effect of diabetes. Infection (*INFEC*) and diabetes (*DIAB*) are flag variables taking the value of 1 in case of infection or diabetes and 0 if otherwise. η_xxi_ represents the IIV. CL, clearance; F1, relative bioavailability; *K_a_*, absorption rate constant; RV, residual variability; SAEM, stochastic approximation of expectation-maximization; SE, standard error; TV, typical volume; *V*_c_, volume of distribution in the central compartment; *V*_p_, volume of distribution in the peripheral compartment.

c CV, coefficient of variation; IIV, interindividual variability; RSE, relative standard error.

d Model parameters were estimated using SAEM from 9,756 PK observations in 1,312 individuals.

e Mean and 95% confidence intervals were generated using only the successful runs (*n *= 432) from a nonparametric bootstrap (*n *= 1,000).

f RSE obtained from the NONMEM R matrix computed on an importance sampling step. The relative SEs for omega and sigma are reported on the approximate standard deviation scale (SE/variance estimate)/2.

g Volumes and CL are reported for a typical individual of 77.3 kg.

h Fold increase on the square root scale.

The model predictive performance was satisfactory as assessed by prediction-corrected visual predictive checks (VPCs) and included stratification by route of administration and/or trial (see Table A3 and Fig. A2 in the supplemental material). An automatic binning method was used, and the model was deemed qualified to simulate tedizolid exposure in the adolescent population. The estimated individual exposure distributions after the first and last dose of tedizolid phosphate in adolescents with ABSSSIs in the PN012 trial were slightly higher but overlapped largely with those in adults with ABSSSIs in the previous phase 2 and 3 trials due to a body weight effect (Table 2). A comparison of tedizolid exposure in adolescents with ABSSSIs based on body weight quartiles showed a small increase in tedizolid exposure with decreasing body weight (Table 3).

**TABLE 2:** Comparison of popPK model-based exposures among participants with ABSSSIs^*a*^

Population	Participants (*n*)	GM^*b*^ (95% CI) of:			
		AUC_0–24h_day1_ (μg·h/ml)	AUC_0–24h_last_ (μg·h/ml)	*C*_max 0–24h_day1_ (μg/ml)	*C*_max 0–24h_last_ (μg/ml)
Adult^*c*^	830	22.4 (21.9, 22.9)	21.0 (20.4, 21.5)	1.81 (1.73, 1.90)	2.00 (1.94, 2.06)
Adolescent^*d*^	91	26.6 (24.9, 28.4)	28.6 (26.6, 30.8)	2.61 (2.27, 3.01)	3.13 (2.89, 3.38)

a popPK, population pharmacokinetics; ABSSSI, acute bacterial skin and skin structure infection.

b AUC_0–24h_day1_, area under the concentration-time curve from 0 to 24 h on day 1; AUC_0–24h_last_, area under the concentration-time curve from 0 to 24 h on the last dosing day; *C*_max 0–24h_day1_, maximum concentration of drug in plasma from 0 to 24 h on day 1; *C*_max 0–24h_last_, maximum concentration of drug in plasma from 0 to 24 h on the last dosing day; GM, geometric mean.

c Participants aged ≥18 years with ABSSSIs who received 200 mg of tedizolid phosphate in the previous phase 2 and 3 trials.

d Participants aged ≥12 years in the phase 3 clinical trial (PN012).

**TABLE 3:** Exposure by weight quartile in adolescents with ABSSSIs in the phase 3 PN012 trial^*a*^

Body wt (kg)	Participants (*n*)	GM (95% CI) of^*b*^:			
		AUC_0–24h_day1_ (μg·h/ml)	AUC_0–24h_last_ (μg·h/ml)	*C*_max 0–24h_day1_ (μg/ml)	*C*_max 0–24h_last_ (μg/ml)
27.6 to 46.5	23	32.2 (29.3, 35.4)	32.4 (29.7, 35.4)	3.32 (2.59, 4.25)	3.87 (3.43, 4.37)
46.5 to 57	23	29.6 (25.7, 34.0)	32.2 (26.4, 39.4)	3.83 (3.45, 4.25)	3.54 (2.93, 4.29)
57 to 70^*c*^	24	25.5 (22.7, 28.7)	27.9 (24.4, 32.0)	2.52 (1.98, 3.20)	2.97 (2.67, 3.30)
70 to 126^*c*^	21	20.1 (18.1, 22.3)	22.6 (20.0, 25.7)	1.38 (0.965, 1.96)	2.29 (2.00, 2.63)

a ABSSSI, acute bacterial skin and skin structure infection.

b AUC_0–24h_day1_, area under the concentration-time curve from 0 to 24 h on day 1; AUC_0–24h_last_, area under the concentration-time curve from 0 to 24 h on the last dosing day; *C*_max 0–24h_day1_, maximum concentration of drug in plasma from 0 to 24 h on day 1; *C*_max 0–24h_last_, maximum concentration of drug in plasma from 0 to 24 h on the last dosing day; GM, geometric mean.

c The imbalanced group sizes were due to 4 participants with a body weight of 70 kg.

### Probability of target attainment.

**FIG 1:**
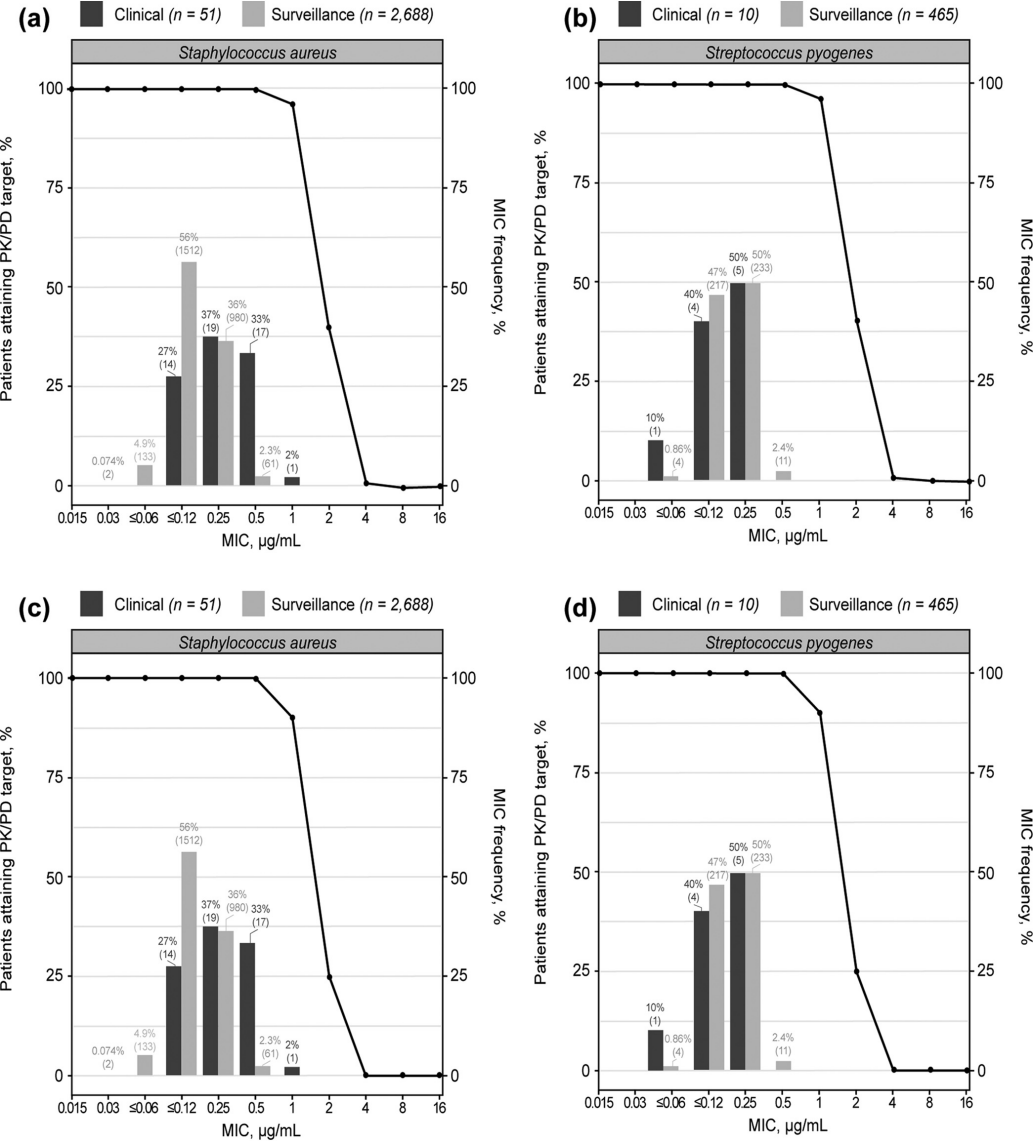
Proportion of participants achieving tedizolid *f*AUC/MIC of ≥3 in the phase 3 PN012 trial in adolescents and global surveillance studies after administration of 200 mg of tedizolid phosphate once daily for 6 days by intravenous (a and b) and oral (c and d) routes. *f*AUC, area under the concentration-time curve for the free, unbound fraction of a drug; PK/PD, pharmacokinetic/pharmacodynamic.

The simulation analyses used the final popPK model of tedizolid to confirm the appropriateness of the proposed dosing regimen by determining the probability of tedizolid PK/PD target attainment. Based on a comparison of body weight distribution between simulated and actual participants in PN012, the National Health and Nutrition Examination Survey database was considered appropriate for use in the adolescent PTA simulations (Fig. A3). With i.v. administration, PTA was 100% over the MIC range of 0.0015 to 0.5 μg/ml (determined from PN012 and surveillance studies; data on file) and dropped to 96.2%, 40.3%, and 1.0% at MICs of 1, 2, and 4 μg/ml, respectively (Fig. 1a and b). Similar results were observed with oral administration; PTA was 100% over the MIC range of 0.0015 to 0.25 μg/ml and dropped to 99.8%, 90.0%, 24.8%, and 0.1% at MICs of 0.5, 1, 2, and 4 μg/ml, respectively (Fig. 1c and d). Therefore, it is predicted that the 200-mg dose of tedizolid phosphate will have ∼100% PTA in adolescents up to the MIC susceptibility breakpoint of 0.5 μg/ml for Staphylococcus and Streptococcus sp.

### Exposure-efficacy analysis.

**FIG 2:**
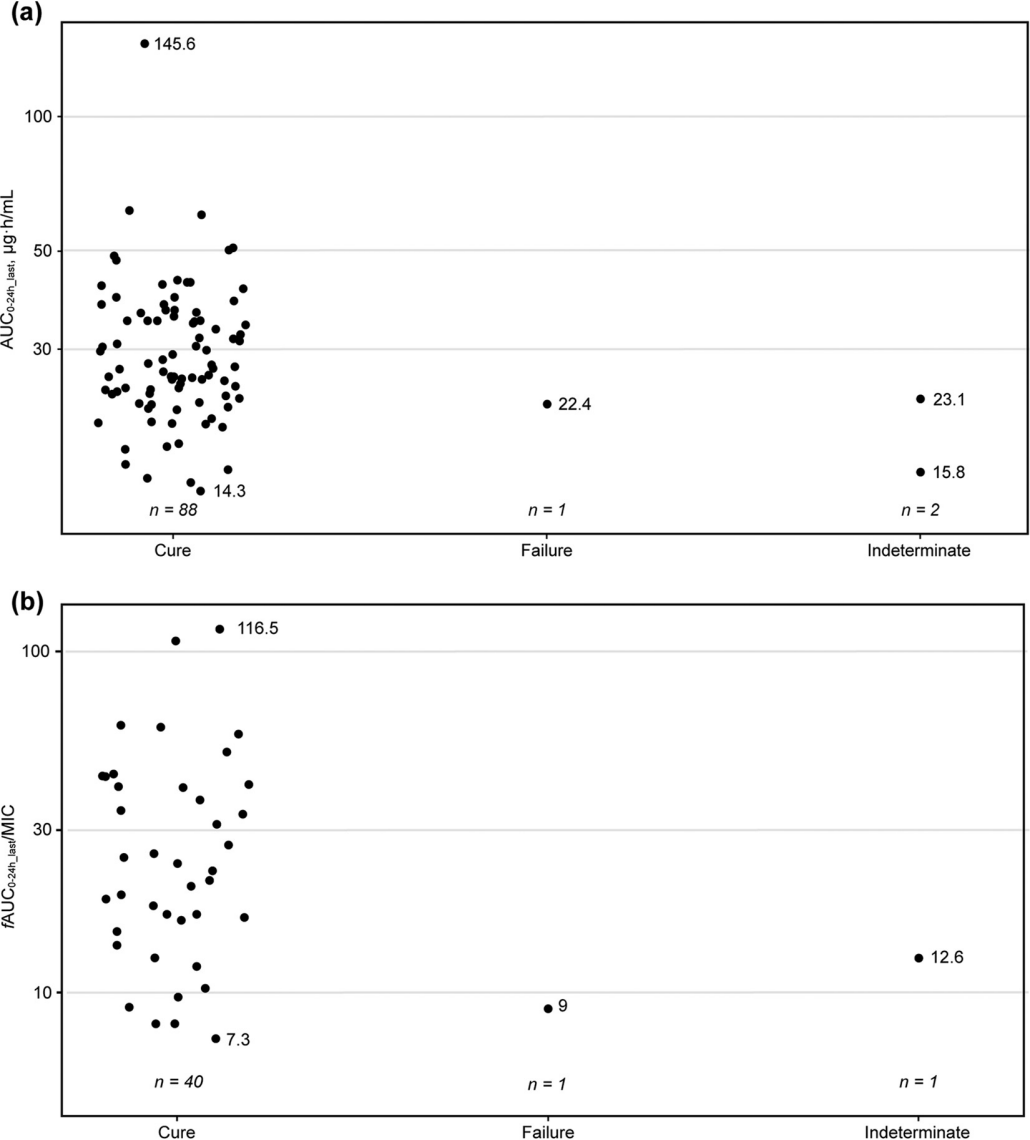
Distribution of predicted tedizolid AUC_0–24h_last_ (a) and *f*AUC_0–24h_last_/MIC (b) in log scale with overall response between cure, failure, and indeterminate at test of cure in the phase 3 PN012 trial in adolescents.^a^ AUC_0–24h_last_, area under the concentration-time curve from 0 to 24 hours on the last dosing day; *f*AUC_0–24h_last_, area under the concentration-time curve for the free, unbound fraction of the drug from 0 to 24 hours on the last dosing day; MIC, MIC. ^a^Pharmacokinetic and microbiological data were not available for every participant.

Among the 91 adolescents who received tedizolid phosphate in PN012, the clinical response at the test-of-cure (TOC) visit was cure in 88 (96.7%), failure in 1 (1.1%), and indeterminate in 2 (2.2%). The exposure-efficacy analysis used clinical response at TOC as the efficacy analysis endpoint. The PK endpoint for each participant was the *f*AUC from 0 to 24 hours on the last dosing day (*f*AUC_0–24h_last_)/MIC and AUC_0–24h_last_ (where *f*AUC was estimated using the popPK model). For the analysis with *f*AUC_0–24h_last_/MIC, 42 of 91 participants had available PK and microbiological data. Graphical comparisons depicted AUC distribution in participants with versus without a favorable clinical response at TOC; participants who were unable to achieve a favorable clinical response had AUC values within the AUC distribution of those who did achieve a clinical response (Fig. 2a). Moreover, no clear trend was observed between tedizolid *f*AUC_0–24h_last_/MIC and efficacy (Fig. 2b), illustrating the lack of a relationship between the PK/PD index of tedizolid at the 200-mg dose and below the baseline pathogen MIC susceptibility breakpoint and clinical response at TOC in adolescents with ABSSSIs.

### Exposure-safety analysis.

**FIG 3:**
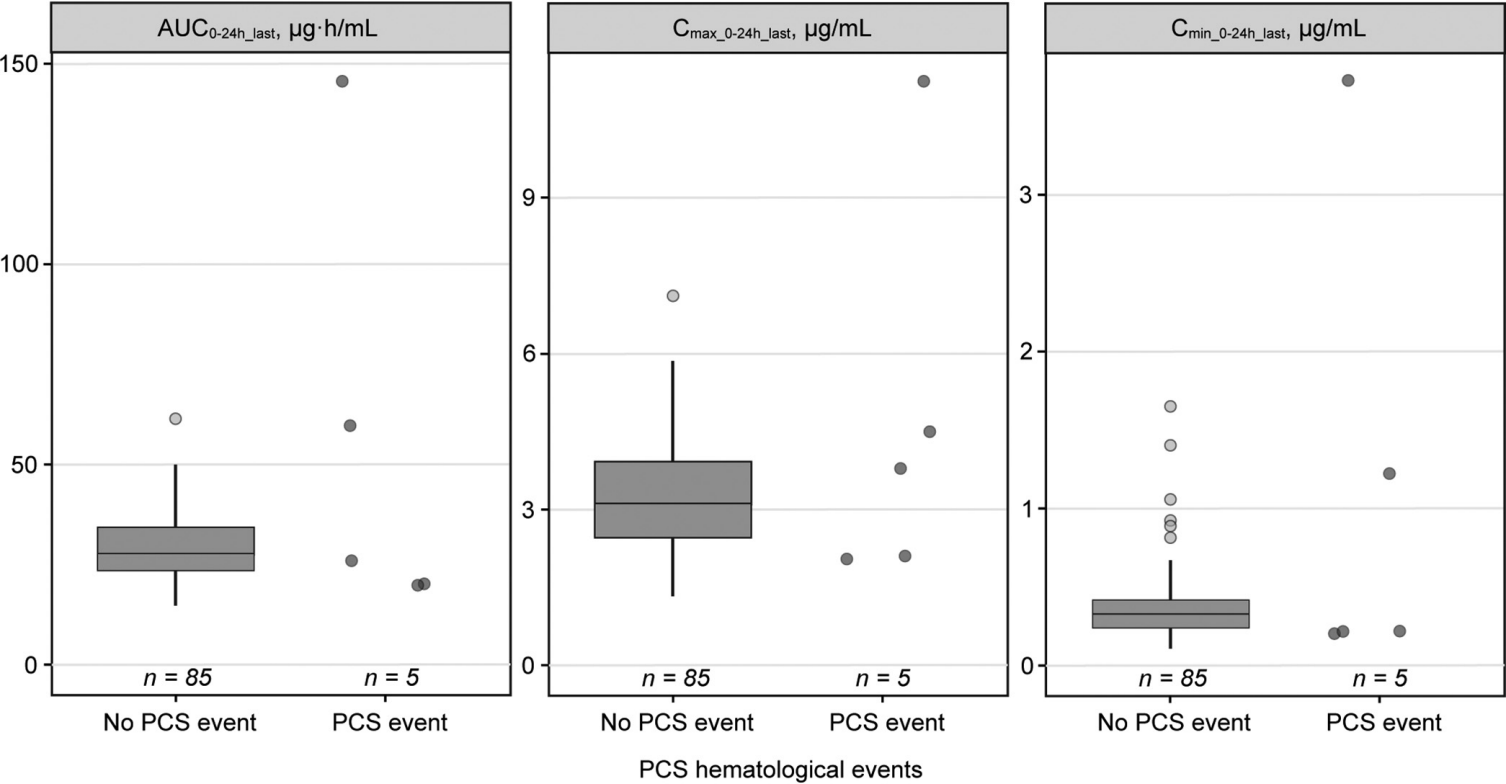
Distribution of tedizolid exposure in adolescents with versus without PCS abnormal hematology values in the phase 3 PN012 trial in adolescents.^a,b^. AUC_0–24h_last_, area under the concentration–time curve from 0 to 24 hours on the last dosing day; *C*_max 0–24h_last_, maximum concentration of drug in plasma from 0 to 24 hours on the last dosing day; *C*_min 0–24h_last_, minimum concentration of drug in plasma from 0 to 24 hours on the last dosing day; PCS, potentially clinically significant. ^a^The participant with *C*_max_ of >9 μg/ml had only one pharmacokinetic data point available to estimate tedizolid exposure. One participant had missing values and could not be evaluated. ^b^The participant with the highest predicted AUC_0–24h_last_ had very high tedizolid plasma concentrations that were mostly excluded as outliers. The AUC_0–24h_last_ for this participant was predicted based on a single concentration measurement after outlier exclusions and should therefore be interpreted with caution.

In PN012, 200 mg of tedizolid phosphate once daily demonstrated an acceptable safety profile. One participant had anemia that was not considered to be drug related; no thrombocytopenia or abnormal neurologic or visual acuity events were observed (10). Abnormal laboratory values considered potentially clinically significant (PCS) were examined for association with tedizolid exposure. Five PCS hematologic events (5.6%) were reported, of which all were related to a decreased absolute neutrophil count. The distribution of exposures (area under the concentration-time curve from 0 to 24 hours on the last dosing day [AUC_0–24h_last_], maximum concentration of drug in plasma from 0 to 24 hours on the last dosing day [*C*_max 0–24h_last_], and minimum concentration of drug in plasma from 0 to 24 hours on the last dosing day [*C*_min 0–24h_last_]) among adolescents who experienced a PCS decrease in absolute neutrophil count was comparable to the distribution of exposures in patients without an event (*n *= 85 [94.4%]) (Fig. 3). One participant had missing values and could not be evaluated. No visible trends in PCS hematologic events were observed across AUC_0–24h_last_ quartiles.

## DISCUSSION

The previous popPK model for tedizolid, developed based on adult data and limited adolescent data and derived using well-established modeling methods, was the starting point for the current analysis (21). The updated model now includes PK data from 16 clinical trials, including the phase 3 PN012 trial in adolescents 12 to <18 years of age (10) and the phase 1 PN013 trial in children 2 to <12 years of age that was ongoing at the time of the analysis (22). Only limited adjustments were made to arrive at the current model, as described in the Materials and Methods section, and the updated model displayed good predictive performance for all trials included; thus, no additional covariate screening was conducted. The updated popPK parameters and covariates were comparable to those estimated using previous PK models (21, 23). The model was rerun using alternative allometric elements, and there was no change in the overall findings of the model. Consistent with the interim model that evaluated PK parameters in children 6 to <12 years of age (23), body weight remained an influential covariate in this updated model, leading to an exposure difference of approximately 30% between the lower and higher quartiles. However, body weight did not have a clinically relevant effect, as no relationship between tedizolid exposure and efficacy or safety and no clinically meaningful difference in tedizolid PK regarding body weight was observed, which is consistent with findings in adults (21). Because the PN012 trial enrolled participants with ABSSSIs and there were no adolescent participants with diabetes, the effect of diabetes on the efficacy and safety in the adolescent population cannot be evaluated directly. However, tedizolid exposure and diabetes were not identified as predictors of clinical response in adults because diabetes was identified only as a significant covariate on central volume but not clearance. Thus, diabetes had no impact on AUC and AUC/MIC, which is the PK index related to efficacy. A clinically meaningful impact of diabetes is not expected in adolescents based on the similar PK between adolescents and adults (21).

Overall, this model was considered suitable for generating individual model-predicted exposures in the adolescents who participated in PN012 and to simulate exposures in the adolescent population. The simulations predicted tedizolid to have 100% PTA based on an *f*AUC/MIC ratio of ≥3 up to the MIC susceptibility breakpoint of 0.5 μg/ml, which is the current breakpoint for both Staphylococcus and Streptococcus sp. (24, 25). At an MIC of 1 μg/ml, PTA was ≥90% for i.v. or orally administered tedizolid (10). Notably, the S. aureus MIC required to inhibit the growth of 90% of isolates from PN012 and from the Surveillance of Tedizolid Activity and Resistance data that examined approximately 7,800 S. aureus isolates collected in the United States and Europe from 2009 to 2013 were both 0.5 μg/ml (5).

A lack of efficacy was not related to exposure, as all study doses were deemed sufficient to provide a response, and no exposure-efficacy relationship was identified. This finding may be explained by the high cure rate (96.7%) among participants from PN012 who received tedizolid phosphate. Exposures were similar between participants with and without a safety event, and no clear relationship was detected between tedizolid exposure distributions and safety. Safety profiles in adolescents were similar to those of adults, despite the lower body weight and higher exposure in adolescents; hence, there is no body weight cutoff required for once-daily 200-mg dosing in adolescents.

One potential limitation of this analysis was the small sample size. There were also limited data for oral administration, so there is uncertainty around certain parameters (i.e., interindividual variability and coefficient of variation were both large for participants receiving oral administration). In addition, the exposure-efficacy analysis was exploratory as the PN012 trial was not powered for formal efficacy evaluation and a limited number of participants had a clinical failure outcome. The range of exposures was also limited in this population, of whom most had favorable clinical responses. The exposure-safety analysis was also exploratory, and only five participants experienced a PCS laboratory abnormality. However, the low rate of safety events for tedizolid in adolescents is supported by another study of tedizolid PK, safety, and tolerability where comparable exposures to those reported in this study (AUC_0–last_, 21.0 and 25.5 μg·h/ml for oral and i.v. administration, respectively) yielded limited and mild adverse events, with no serious adverse events or related study discontinuations (9). Based on previous clinical experience, there were no safety concerns at the approved dose level. In the phase 1 study in adolescent patients, tedizolid phosphate administered as a single oral or i.v. dose of 200 mg was generally well tolerated (9). A strength of this study is the inclusion of pediatric data from multiple clinical trials in the updated popPK model; the variability in demographics (including body weight) in this population may be more reflective of a real-world patient population. Inclusion of hospitalized pediatric participants further enhances the appropriateness of the updated model.

In summary, tedizolid exposure in adolescent patients with ABSSSIs after i.v. or oral administration was generally similar to exposure in adult patients with ABSSSIs. High PTA was achieved in adolescents after i.v. and oral administration of 200 mg of tedizolid phosphate up to the MIC susceptibility breakpoint of 0.5 μg/ml. Although body weight impacted exposure, no significant dose-response relationship was evident, and no additional safety concerns in the adolescent population were identified. The 200-mg dose in adolescent patients is further supported by the safety and efficacy studies that have been conducted in adult and pediatric participants (7, 8, 10). Therefore, the 200-mg once-daily dose of tedizolid phosphate is appropriate for the treatment of ABSSSIs in adolescent patients.

## MATERIALS AND METHODS

### Data selection.

The analysis data set included newly obtained PK data from two clinical trials, as follows: (i) a randomized, single-blind, multicenter, phase 3 trial that evaluated the efficacy and safety of tedizolid phosphate in adolescents 12 to <18 years of age with ABSSSIs (ClinicalTrials.gov identifier NCT02276482 [protocol MK-1986-012]; here referred to as PN012) (10) and (ii) a phase 1, single-dose PK and safety trial of oral and i.v. tedizolid phosphate in hospitalized children 2 to <12 years of age receiving treatment or prophylaxis for Gram-positive infection (NCT02750761 [protocol MK-1986-013]; here referred to as PN013). The PN013 trial was ongoing at the time of model development (22). All trials were conducted in accordance with the principles of Good Clinical Practice and were approved by the appropriate institutional review boards and regulatory agencies. PK data from these trials were combined with the data set used to construct an early popPK model, including a phase 1 PK trial in adolescents (MK-1986-026) (21) and a phase 3 trial in adults and adolescents (MK-1986-010) (7). Overall, data from 16 trials were included in this current model (Tables A1 and A2). Covariate evaluation was conducted in the initial model and utilized a stepwise forward-selection/backward-elimination procedure, as described previously (21). Across all covariates in the early model, only ideal body weight (for clearance and central volume) and total bilirubin (for clearance) emerged as significant predictors of tedizolid PK. In an interim model that included 1,160 adults and 41 pediatric participants, body weight (all parameters except intercompartmental clearance), ABSSSIs (clearance and central volume), and diabetes (central volume) emerged as significant predictors for tedizolid PK in pediatric patients aged <12 years (23).

### PopPK model development.

The popPK analysis was performed using a nonlinear mixed-effects modeling approach. The applied estimation methods in NONMEM version 7.3.0 (ICON, Hanover, MD) were first-order conditional estimation with interaction, followed by stochastic approximation expectation maximization for the parameter estimation; first-order conditional estimation was used for the bootstrap analysis. The previously developed popPK model, which consisted of a two-compartment model with linear elimination, a sequential zero-order release into a depot compartment, and first-order sigmoidal absorption from the depot to the central compartment (21), provided the backbone of the current model. The popPK model for tedizolid was updated, and covariates were reevaluated several times using accumulating data from different populations (Japanese patients, Chinese patients, and patients with hospital-acquired bacterial pneumonia/ventilator-associated bacterial pneumonia [HABP/VABP]). Covariates including ideal body weight and bilirubin were removed and covariates including diabetes, infection, and body weight were added during the interim model updates (unpublished). Only limited adjustments were made to arrive at the current model, and they included (i) a change in the reference population for the effect of disease on clearance and volume of distribution in the central compartment (*V*_c_) from patients with ABSSSI to healthy volunteers and (ii) fixing the exponent of the power effect of weight on volume of distribution (*V*_c_ and volume of distribution in the peripheral compartment) and clearance (clearance and intercompartmental clearance) parameters to better characterize PK across the weight range in adolescent patients. Parameters were reestimated using the updated data set from the adolescent and pediatric populations. Individual concentration data points were classified as potential outliers based on conditional weighted residuals; those with conditional weighted residuals of >6 were excluded. After reestimation, the outliers were considered influential and remained excluded if any of the parameter estimates had shifted by ≥10%. The exclusion and reestimation process was conducted iteratively until all influential outliers were excluded.

The stability and robustness of the final model were assessed through prediction-corrected VPCs and bootstrap analysis. For the prediction-corrected VPC, 500 simulations of the original data set were performed using the final model. The 95% confidence interval of the simulated median and 5th and 95th percentiles were plotted with observed data overlaid to evaluate the model performance. Concordance between simulated and observed quantiles was used to qualify the model as suitable to predict concentration-time profiles. A nonparametric bootstrap analysis was conducted to obtain parameter uncertainty estimates (first-order conditional estimation). For bootstrap analysis, 1,000 replicate data sets were obtained by randomly resampling participants from the original data set with the replacement data set. The final model was refitted on each of the resampled data sets, and parameter estimates from the runs with successful minimization were summarized as nonparametric distribution intervals across replicates. The final popPK model was used to predict the individual exposure parameters for adolescent participants to support the dosing recommendations for tedizolid phosphate in this population. Full PK profiles of tedizolid plasma concentration were estimated for each participant based on the individual empirical Bayesian estimates and their actual treatment regimen. The estimated exposures were compared graphically with adult exposure distributions (see Fig. A4 in the supplemental material). The *post hoc* estimates of PK parameters were further used in the following exposure-efficacy and exposure-safety analyses.

### PopPK/PD analyses.

**Probability of target attainment analysis.** Using the final popPK model, tedizolid PK profiles were generated by Monte Carlo simulation; 1,000 adolescent patients were simulated to receive 200 mg of tedizolid phosphate administered i.v. or orally once daily for 6 days. Demographic and body weight data used for the simulation were sampled randomly from corresponding age ranges in the National Health and Nutrition Examination Survey database curated by the US Centers for Disease Control and Prevention (26). Tedizolid concentration was quantitated using high-performance liquid chromatography, and the concentration of free, unbound tedizolid was calculated using a correction for mean protein binding of 87.3% ± 1.3% (15). *f*AUC/MIC was calculated for each simulated patient over a defined range of MIC values to generate PTA curves. The PTA curves depicted the percentage of simulated patients achieving the PK/PD target of an *f*AUC/MIC of ≥3, which corresponded to stasis to a 1-log kill of S. aureus in a nonneutropenic murine thigh infection model (18–20). PTA curves were overlaid with MIC distributions for S. aureus and Streptococcus pyogenes obtained from PN012 and surveillance studies (data on file) and evaluated graphically. Isolates were identified locally and forwarded to a central monitoring laboratory (JMI Laboratories, North Liberty, IA), where they were retested for identity and the MICs were determined.

### Exposure-efficacy analysis.

The exploratory exposure-efficacy analysis used efficacy data from PN012 (10), a secondary endpoint of which was the clinical response at the TOC visit (18 to 25 days or 25 days after the first infusion) in the intention-to-treat population. The relationship between the individual AUC or *f*AUC/MIC and the clinical outcomes was examined graphically. Individual isolate MIC values from the trial, obtained at baseline screening, were used in the analysis. AUC and *f*AUC/MIC were compared for participants with versus without a favorable clinical response at TOC to assess any shift in PK distribution with response. A favorable clinical response was a ≥20% reduction from the baseline lesion area (defined as length × width of the erythema, edema, and/or induration). The proportion of participants that achieved a favorable response was assessed by AUC and *f*AUC/MIC quartile to evaluate potential trends between exposure and efficacy outcomes.

### Exposure-safety analysis.

The exploratory exposure-safety analysis used safety data from the tedizolid phosphate treatment arm of the PN012 trial and focused on events that could be associated with the oxazolidinone class of antibacterials, namely, changes in hematologic parameters and optic and peripheral neuropathy (10). A graphical exploration of the exposure-response relationship was planned if ≥5 participants who received tedizolid phosphate in PN012 experienced neurologic or visual acuity events. PK parameters for tedizolid, including AUC_0–24h_last_, *C*_max 0–24h_last_, and *C*_min 0–24h_last_, were compared for participants with versus without a PCS hematologic event. If PCS hematologic events occurred in a sufficient number of participants (*n* ≥ 5), the potential relationship between tedizolid exposure and response was explored. Laboratory values were assessed at screening, day 7, end of treatment, and TOC. Cutoffs for PCS changes in hematology values were defined in the protocol. For participants with normal laboratory values and those with baseline values below the lower limit of normal (LLN), PCS abnormal laboratory values included postbaseline platelet or hemoglobin levels at <75% of the LLN or of baseline, respectively, and neutrophil counts at <50% of the LLN or of baseline, respectively. Neurologic and visual acuity examinations were conducted throughout the trial.

### Data availability.

The data sharing policy of Merck Sharp & Dohme Corp., a subsidiary of Merck & Co., Inc., Kenilworth, NJ, USA (MCD), including restrictions, is available online at http://engagezone.msd.com/ds_documentation.php. Requests for access to the clinical study data can be submitted through the EngageZone site or via email to dataaccess@merck.com.
