# Supplementary material for: Population Pharmacokinetics, Exposure-Response, and Probability of Target Attainment Analyses for Tedizolid in Adolescent Patients with Acute Bacterial Skin and Skin Structure Infections
Source: Antimicrob Agents Chemother. 2021 Nov 17;65(12):e00895-21. doi: 10.1128/AAC.00895-21 (PMC8597753; doi:10.1128/AAC.00895-21)

APPENDIX

SUPPLEMENTARY INFORMATION

Table A1. Summary statistics of categorical covariates by trial

Table A2. Analysis data set: summary statistics of continuous covariates by trial

Table A3. Distribution of PK samples stratified by route of administration and age

FIG. A1. Goodness-of-fit plots. CWRES, conditional weighted residuals; DV, dependent variable; IPRED, individual predicted concentration; IWRES, individual weighted residuals; PRED, population model predictions; TSLD, time since last dose.

FIG. A2. Prediction-corrected VPC of the final model for all trials.

Note: Within each panel, the median (bold line) and 5th and 95th percentiles (dashed lines) of the observed data are compared to the 95% confidence intervals (shaded areas) for the median (dark area) and 5th and 95th percentiles of the simulated (*n* = 500) data (light areas). Individual observations (*n* = 9,756) are not shown for clarity.

VPC, visual predictive check.

[a]Oral and IV administration or IV to oral switch permitted.

[b]Oral administration only.

FIG. A3. Comparison of body weight distribution between simulated and actual adolescent participants.

Note: The simulated data were sampled from the National Health and Nutrition Examination Survey data set in participants 12 to <18

years of age; the observed data are participants in the phase 3 adolescent trial. The box is delimited by the 25th, 50th, and 75th percentiles; the whiskers extend to the lowest and largest values but no further than 1.5 times the interquartile range from the hinge. The dots beyond the whiskers are outliers and are represented individually.

n, number of participants.

FIG. A4. Box plots of popPK model predicted AUC after last dose in adolescent (study PN012) and adult participants with ABSSSIs.

Note: The adult population includes participants with ABSSSIs >18 years old receiving 200 mg of tedizolid phosphate from previous Phase 2 and 3 studies. The boxes are delimited by the 25th, 50th, and 75th percentiles; the whiskers extend to the lowest and largest values but no further than 1.5 times interquartile range from the hinge. The dots beyond the whiskers are outliers and are represented individually.

ABSSSI, acute bacterial skin and skin structure infection; AUC, area under the concentration-time curve; $AUC_{0–24h\_last}$, area under the concentration–time curve from 0 to 24 hours on the last dosing day; popPK, population pharmacokinetics.

Table A1. Summary statistics of categorical covariates by trial

Covariate	Trial PN013 (*n* = 21)	Trial PN007 (*n* = 175)	Trial PN008 (*n* = 61)	Trial PN028 (*n* = 28)	Trial PN026 (*n* = 20)	Trial PN009 (*n* = 328)	Trial PN010 (*n* = 320)	Trial PN027 (*n* = 20)	Trial PN012 (*n* = 91)
Sex									
Male	15 (71.4)	115 (65.7)	44 (72.1)	14 (50.0)	16 (80.0)	201 (61.3)	218 (68.1)	17 (85.0)	58 (63.7)
Female	6 (28.6)	60 (34.3)	17 (27.9)	14 (50.0)	4 (20.0)	127 (38.7)	102 (31.9)	3 (15.0)	33 (36.3)
Race									
White	16 (76.2)	132 (75.4)	50 (82.0)	28 (100.0)	17 (85.0)	277 (84.5)	278 (86.9)	15 (75.0)	80 (87.9)
Asian	—	1 (0.6)	—	—	1 (5.0)	2 (0.6)	4 (1.2)	—	—
Black	4 (19.0)	40 (22.9)	9 (14.8)	—	1 (5.0)	38 (11.6)	34 (10.6)	5 (25.0)	11 (12.1)
Other	1 (4.8)	2 (1.1)	2 (3.3)	—	1 (5.0)	11 (3.4)	4 (1.2)	—	—
Participant condition									
Healthy volunteer	—	—	61 (100.0)	28 (100.0)	—	—	—	20 (100.0)	—
ABSSSI	—	175 (100.0)	—	—	—	328 (100.0)	320 (100.0)	—	91 (100.0)
Hospitalized	21 (100.0)	—	—	—	20 (100.0)	—	—	—	—
Diabetic status									
Nondiabetic	21 (100.0)	—	61 (100.0)	28 (100.0)	20 (100.0)	302 (92.0)	288 (90.0)	20 (100.0)	91 (100.0)
Diabetic	—	—	—	—	—	26 (7.9)	31 (9.69)	—	—
Missing	—	175 (100.0)	—	—	—	—	1 (0.31)	—	—

Covariate	Trial PN031 (*n* = 24)	Trial PN001 (*n* = 32)	Trial PN005 (*n* = 83)	Trial BAY-16101 (*n* = 26)	Trial BAY-16102 (*n* = 16)	Trial PN006 (*n* = 51)	Trial BAY-16411 (*n* = 16)	Overall (*N* = 1,312)
Sex								
Male	15 (62.5)	24 (75.0)	54 (65.1)	26 (100.0)	16 (100.0)	35 (68.6)	16 (100.0)	884 (67.4)
Female	9 (37.5)	8 (25.0)	29 (34.9)	—	—	16 (31.4)	—	428 (32.6)
Race								
White	11 (45.8)	26 (81.2)	—	—	—	—	—	930 (70.9)
Asian	—	—	83 (100.0)	26 (100.0)	16 (100.0)	51 (100.0)	16 (100.0)	200 (15.2)
Black	12 (50.0)	5 (15.6)	—	—	—	—	—	159 (12.1)
Other	1 (4.2)	1 (3.1)	—	—	—	—	—	23 (1.8)
Participant condition								
Healthy volunteer	24 (100.0)	32 (100.0)	—	26 (100.0)	16 (100.0)	—	16 (100.0)	223 (17.0)
ABSSSI	—	—	83 (100.0)	—	—	51 (100.0)	—	1,048 (79.9)
Hospitalized	—	—	—	—	—	—	—	41 (3.1)
Diabetic status								
Nondiabetic	—	—	46 (55.4)	26 (100.0)	16 (100.0)	44 (86.3)	16 (100.0)	979 (74.6)
Diabetic	—	—	37 (44.6)	—	—	7 (13.7)	—	101 (7.7)
Missing[a]	24 (100.0)	32 (100.0)	—	—	—	—	—	232 (17.7)

ABSSSI, acute bacterial skin and skin structure infection; *n*, number of participants.

[a]If diabetic status was missing in a given study, the value was set to missing (value of –99).

Note: Trial 13 corresponds to PN013, Trial 111 corresponds to PN026, and Trial 122 corresponds to PN012.

Table A2. Analysis data set: summary statistics of continuous covariates by trial

Covariate	Trial PN013 (*n* = 21)	Trial PN007 (*n* = 175)	Trial PN008 (*n* = 61)	Trial PN028 (*n* = 28)	Trial PN026 (*n* = 20)	Trial PN009 (*n* = 328)	Trial PN010 (*n* = 320)	Trial PN027 (*n* = 20)	Trial PN012 (*n* = 91)
Age, y									
Mean (SD)	6.95 (2.40)	36.2 (12.2)	28.6 (8.39)	52.8 (20.4)	14.4 (1.69)	43.7 (15.0)	46.0 (15.8)	28.1 (8.57)	15.0 (1.75)
Median (min, max)	7.00 (3.00, 11.0)	35.0 (18.0, 68.0)	26.0 (18.0, 48.0)	55.5 (25.0, 78.0)	14.0 (11.0, 17.0)	43.5 (18.0, 86.0)	46.0 (17.0, 86.0)	25.0 (20.0, 50.0)	15.0 (12.0, 18.0)
Weight, kg									
Mean (SD)	26.0 (9.06)	81.6 (14.5)	76.3 (12.4)	79.2 (14.3)	60.1 (12.6)	82.3 (18.3)	84.0 (24.4)	82.4 (12.5)	59.7 (17.3)
Median (min, max)	26.9 (12.6, 42.3)	80.0 (47.0, 118)	76.1 (48.9, 111)	79.7 (58.5, 112)	63.5 (38.5, 83.1)	80.3 (47.7, 138)	79.8 (40.5, 226)	82.8 (55.3, 106)	56.6 (27.6, 126)
BMI, kg/m^2									
Mean (SD)	16.3 (2.30)	27.0 (4.37)	25.2 (2.90)	27.7 (3.26)	21.9 (3.41)	27.9 (5.34)	28.5 (7.85)	26.8 (3.31)	21.9 (4.84)
Median (min, max)	15.8 (12.7, 21.0)	26.7 (18.5, 36.0)	25.5 (19.8, 31.5)	27.5 (22.0, 33.6)	22.5 (16.0, 28.4)	27.4 (16.0, 40.0)	26.9 (15.6, 69.9)	26.3 (20.9, 33.4)	20.8 (14.2, 45.0)

Covariate	Trial PN031 (*n* = 24)	Trial PN001 (*n* = 32)	Trial PN005 (*n* = 83)	Trial BAY-16101 (*n* = 26)	Trial BAY-16102 (*n* = 16)	Trial PN006 (*n* = 51)	Trial BAY-16411 (*n* = 16)	Overall (*N* = 1,312)
Age, y								
Mean (SD)	56.4 (9.34)	54.4 (6.98)	63.2 (16.5)	26.9 (4.94)	27.8 (5.09)	48.2 (17.7)	26.2 (5.28)	40.7 (18.2)
Median (min, max)	59.0 (40.0, 74.0)	53.5 (39.0, 67.0)	67.0 (25.0, 94.0)	26.0 (21.0, 37.0)	27.0 (21.0, 35.0)	49.0 (18.0, 79.0)	25.5 (19.0, 36.0)	40.0 (3.00, 94.0)
Weight, kg								
Mean (SD)	83.8 (19.1)	94.1 (19.9)	67.5 (14.8)	64.2 (6.88)	63.2 (7.07)	70.5 (15.3)	62.2 (7.98)	77.6 (21.2)
Median (min, max)	78.8 (49.7, 125)	97.3 (58.6, 151)	67.0 (38.8, 109)	64.3 (53.5, 78.2)	62.9 (50.5, 75.4)	68.0 (47.0, 111)	62.2 (49.7, 76.6)	76.0 (12.6, 226)
BMI, kg/m^2								
Mean (SD)	28.5 (4.73)	31.0 (4.90)	25.8 (5.19)	21.6 (2.05)	21.2 (2.36)	25.1 (4.16)	22.1 (1.87)	26.6 (6.12)
Median (min, max)	27.7 (21.0, 39.6)	31.5 (21.0, 38.9)	25.0 (16.9, 47.9)	21.4 (18.3, 25.5)	20.5 (18.3, 25.1)	24.7 (18.4, 35.9)	22.5 (19.4, 24.7)	25.8 (12.7, 69.9)

BMI, body mass index; max, maximum; min, minimum; SD, standard deviation.

Table A3. **Distribution of PK samples stratified by route of administration and age**

Study	Oral Administration		Intravenous Administration		Number of Evaluable PK Observations[a]	
	PK Samples	BLQ Samples	PK Samples	BLQ Samples	Adult ≥18 y	Pediatric <18 y
PN013	48	-	105	-	-	153[b]
PN007	1,153	-	-	-	1,153	-
PN008	93	-	1,251	-	1,344	-
PN028	385	-	-	-	385	-
PN026	70	10	77	10	-	147[b]
PN009	1,169	-	-	-	1,169	-
PN010	570	-	646	-	1,213	3
PN027	220	-	-	-	220	-
PN012	80	5	350	3	30	400[b]
PN031	-	-	534	-	534	-
PN001	481	-	-	-	481	-
PN005	73	-	353	-	426	-
BAY-16101	87	-	408	-	495	-
BAY-16102	228	-	280	-	508	-
PN006	37	-	212	-	249	-
BAY-16411	452	-	431	-	883	-
Total	5,146	15	4,647	13	9,090	703

BLQ, below the limit of quantitation; PK, pharmacokinetic.

[a]Evaluable PK observations exclude BLQ observations and samples with no measured concentration.

[b]Study 13 only included participants 2 to <12 years of age: study 111 and 122 only included adolescents (11 to 18 years of age).

Note: Study 13 corresponds to PN013, Study 111 corresponds to PN026, and Study 122 corresponds to PN012.

.

FIG A1. Goodness-of-fit plots. CWRES, conditional weighted residuals; DV, dependent variable; IPRED, individual predicted concentration; IWRES, individual weighted residuals; PRED, population model predictions; TSLD, time since last dose.

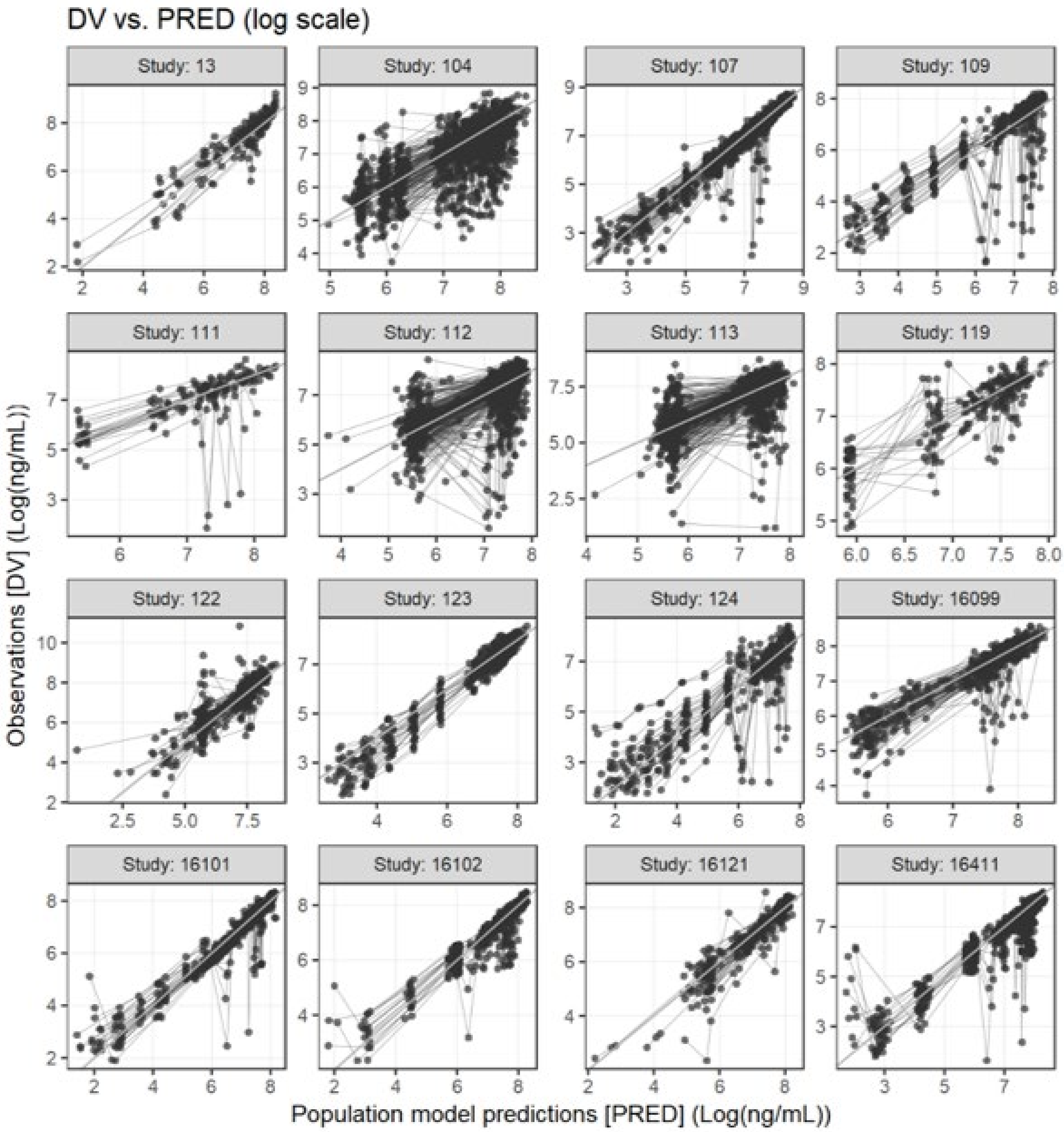

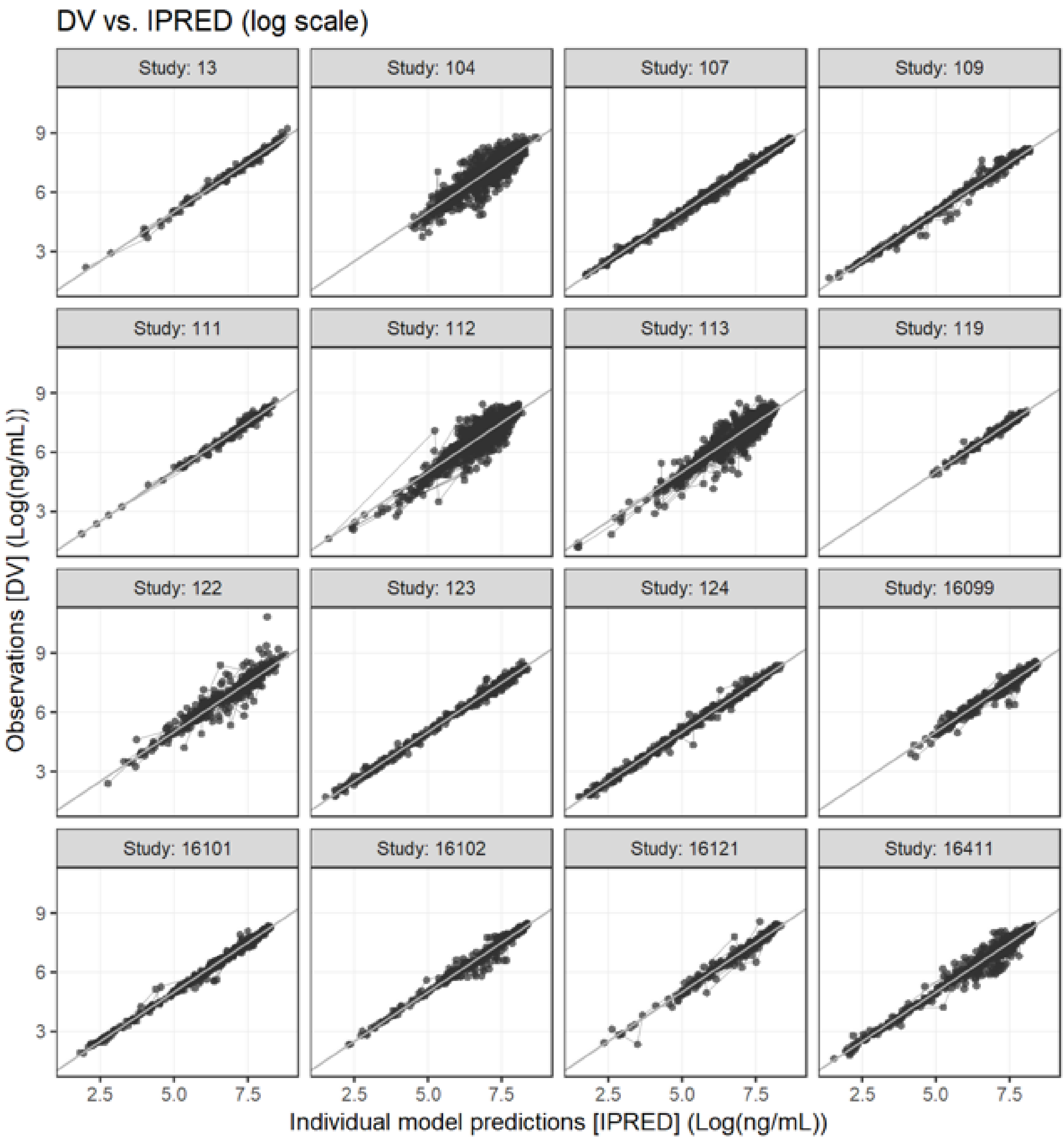
DV vs. IPRED (log scale)
Study: 13
Study: 104
Study: 107
Study: 109
Study: 111
Study: 112
Study: 113
Study: 119
Study: 122
Study: 123
Study: 124
Study: 16099
Study: 16101
Study: 16102
Study: 16121
Study: 16411
Observations [DV] (Log(ng/mL))
Individual model predictions [IPRED] (Log(ng/mL))
3
6
9
2.5
5.0
7.5

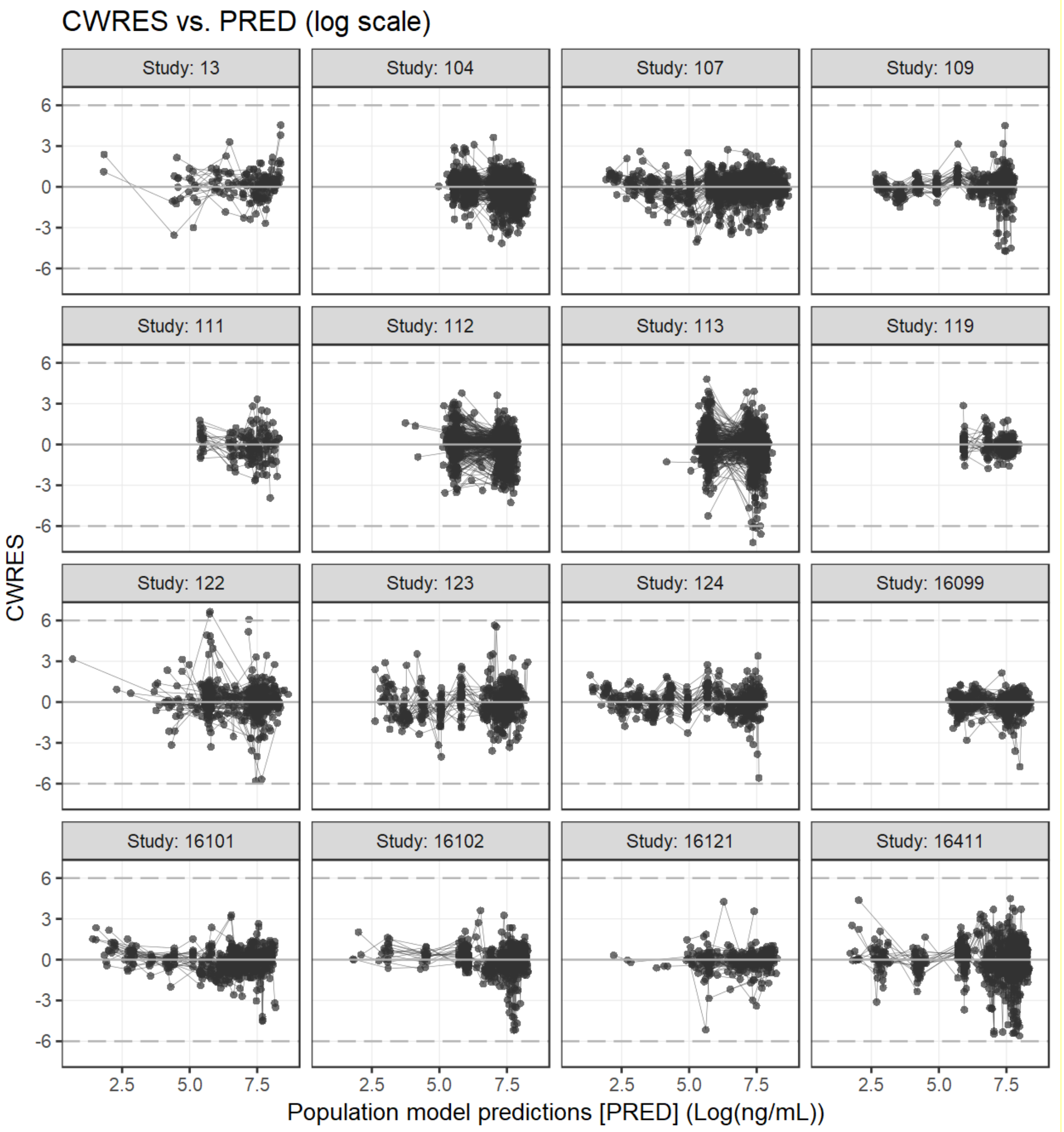
CWRES vs. PRED (log scale)
Study: 13
Study: 104
Study: 107
Study: 109
Study: 111
Study: 112
Study: 113
Study: 119
Study: 122
Study: 123
Study: 124
Study: 16099
Study: 16101
Study: 16102
Study: 16121
Study: 16411
CWRES
6
3
0
-3
-6
2.5
5.0
7.5
Population model predictions [PRED] (Log(ng/mL))

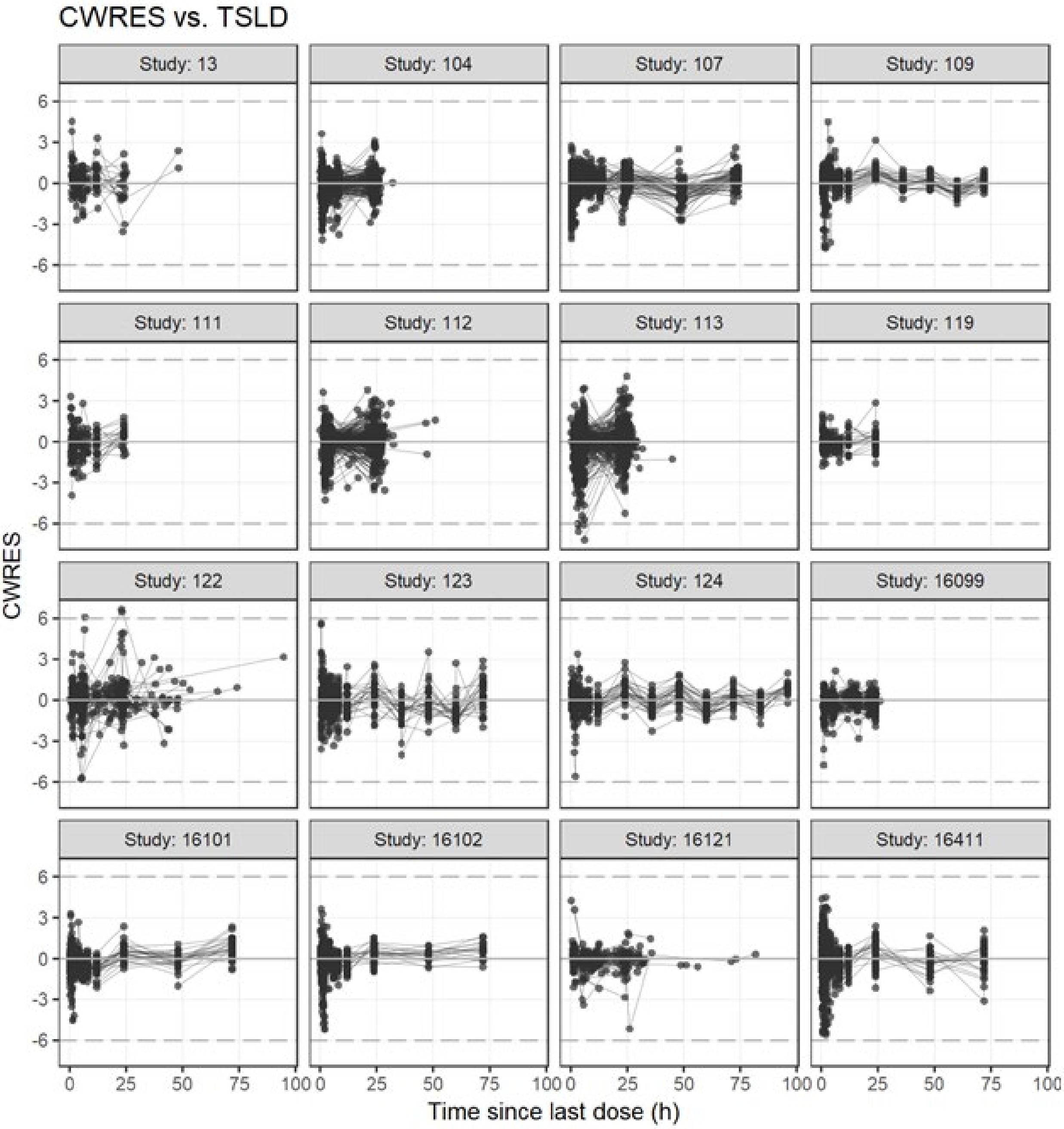

CWRES vs. TSLD
Study: 13
Study: 104
Study: 107
Study: 109
Study: 111
Study: 112
Study: 113
Study: 119
Study: 122
Study: 123
Study: 124
Study: 16099
Study: 16101
Study: 16102
Study: 16121
Study: 16411
CWRES
6
3
0
-3
-6
0
25
50
75
100
Time since last dose (h)

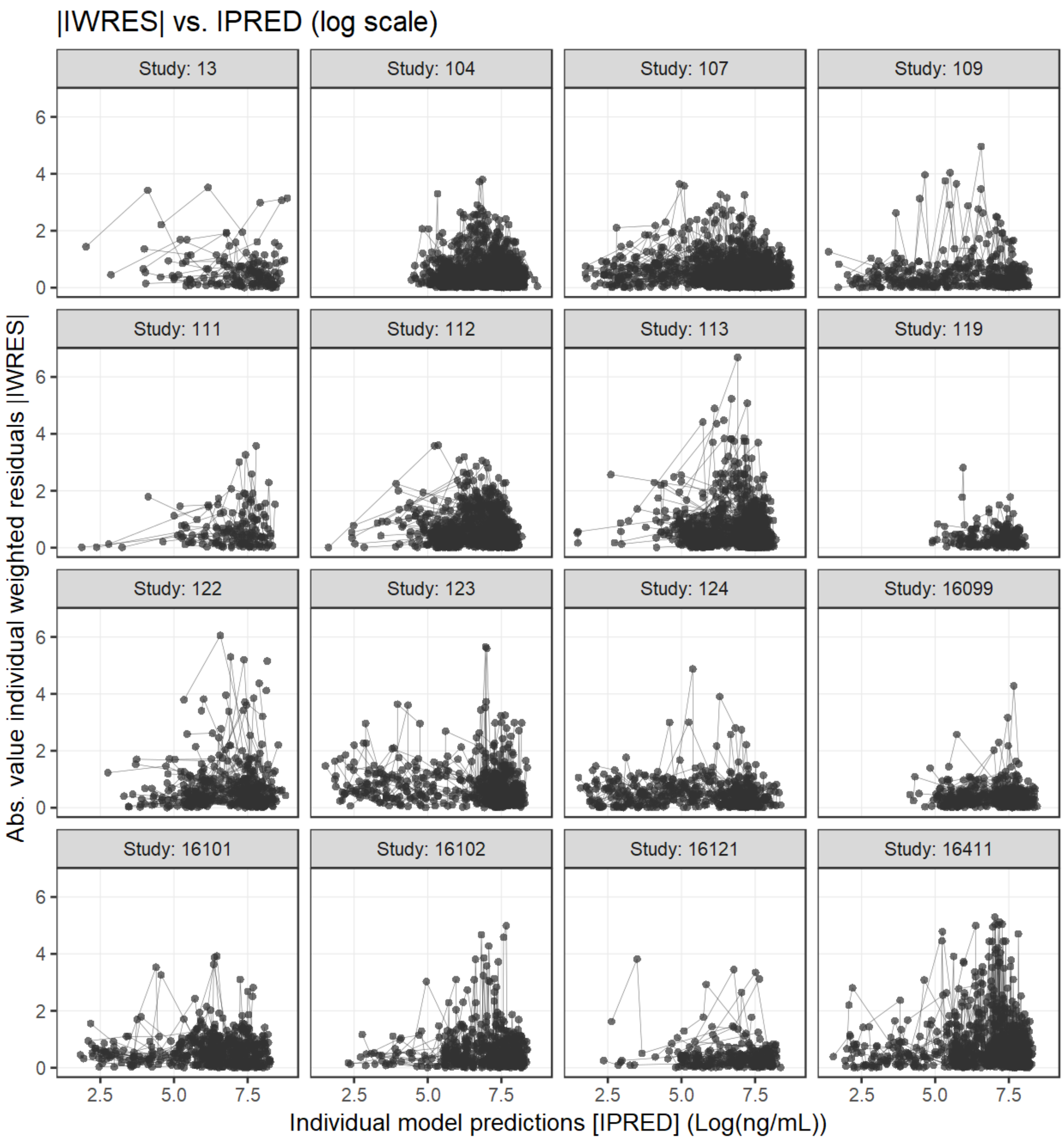
|IWRES| vs. IPRED (log scale)
Study: 13
Study: 104
Study: 107
Study: 109
Study: 111
Study: 112
Study: 113
Study: 119
Study: 122
Study: 123
Study: 124
Study: 16099
Study: 16101
Study: 16102
Study: 16121
Study: 16411
Abs. value individual weighted residuals |IWRES|
Individual model predictions [IPRED] (Log(ng/mL))
0
2
4
6
2.5
5.0
7.5

FIG. A2. Prediction-corrected VPC of the final model for all trials.

Note: Within each panel, the median (bold line) and 5th and 95th percentiles (dashed lines) of the observed data are compared to the 95% confidence intervals (shaded areas) for the median (dark area) and 5th and 95th percentiles of the simulated (n = 500) data (light areas). Individual observations (n = 9,756) are not shown for clarity. The lower limit of quantification was 5 ng/mL.

VPC, visual predictive check.

[a]Oral and IV administration or IV to oral switch permitted.

[b]Oral administration only.

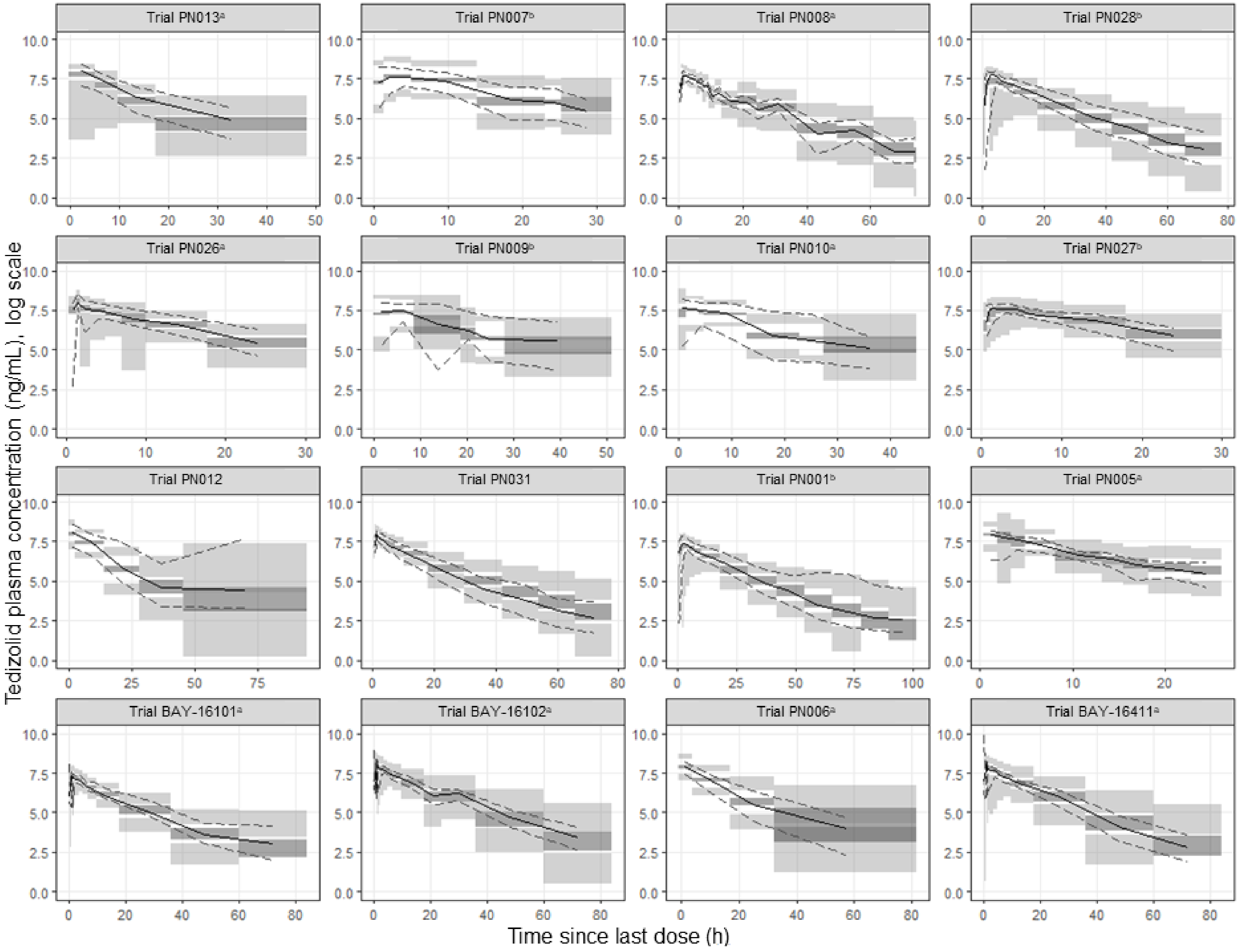
Trial PN013[a]
Trial PN007[b]
Trial PN008[a]
Trial PN028[b]
Trial PN026[a]
Trial PN009[b]
Trial PN010[a]
Trial PN027[b]
Trial PN012
Trial PN031
Trial PN001[b]
Trial PN005[a]
Trial BAY-16101[a]
Trial BAY-16102[a]
Trial PN006[a]
Trial BAY-16411[a]
Tedizolid plasma concentration (ng/mL), log scale
Time since last dose (h)

FIG. A3. Comparison of body weight distribution between simulated and actual adolescent participants.

Note: The simulated data were sampled from the National Health and Nutrition Examination Survey data set in participants 12 to <18 years of age; the observed data are participants in the phase 3 adolescent trial. The box is delimited by the 25th, 50th, and 75th percentiles; the whiskers extend to the lowest and largest values but no further than 1.5 times the interquartile range from the hinge. The dots beyond the whiskers are outliers and are represented individually. *n*, number of participants.

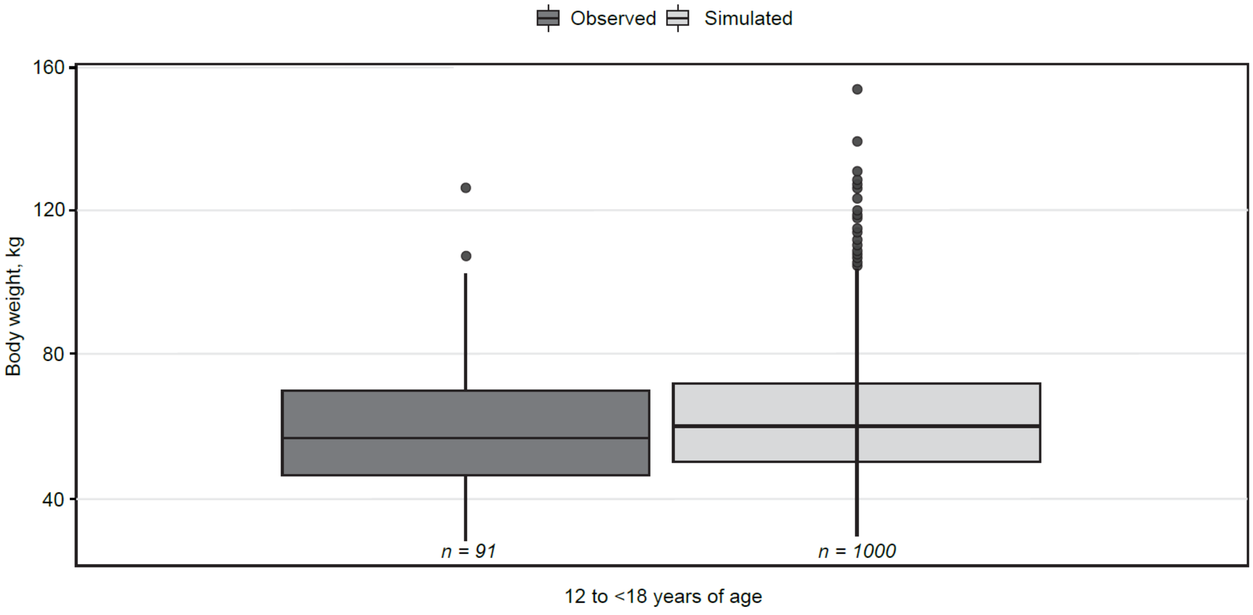

FIG. A4. Box plots of popPK model predicted AUC after last dose in adolescent (study PN012) and adult participants with ABSSSIs.

ABSSSI, acute bacterial skin and skin structure infection; AUC, area under the concentration-time curve; $AUC_{0–24h\_last}$, area under the concentration–time curve from 0 to 24 hours on the last dosing day; popPK, population pharmacokinetics.

Note: The adult population includes participant with ABSSSIs >18 years old receiving 200 mg of tedizolid phosphate from previous Phase 2 and 3 studies. The boxes are delimited by the 25th, 50th, and 75th percentiles; the whiskers extend to the lowest and largest values but no further than 1.5 times interquartile range from the hinge. The dots beyond the whiskers are outliers and are represented individually.

Linear scale

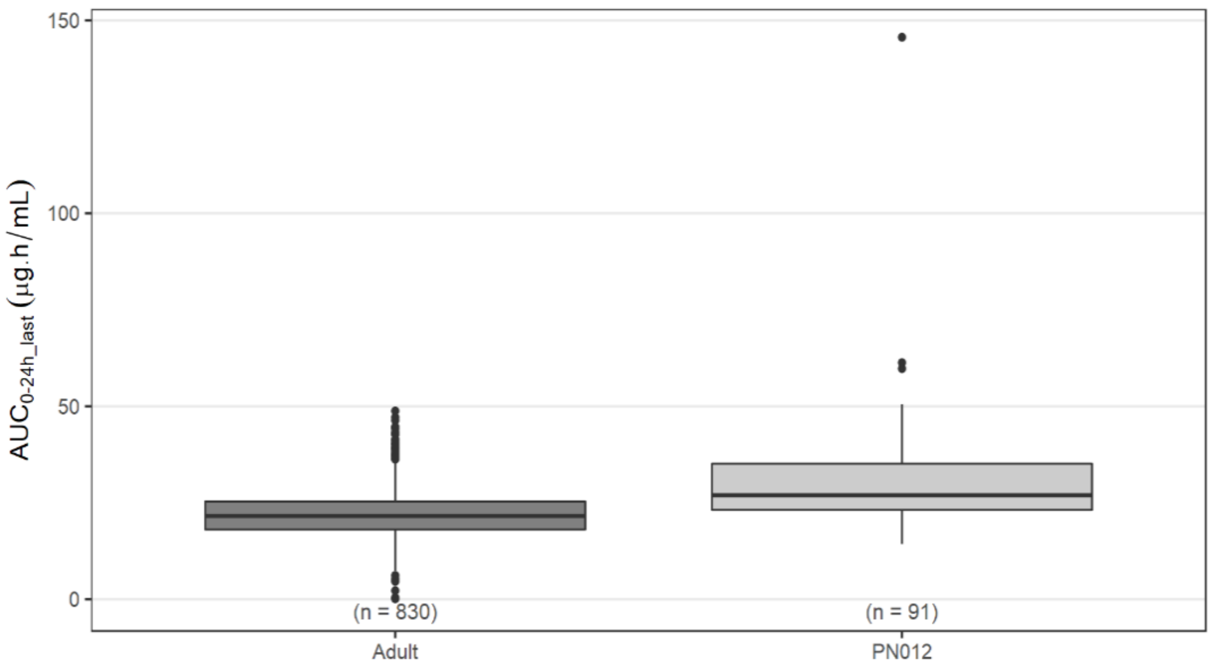

Logarithmic scale

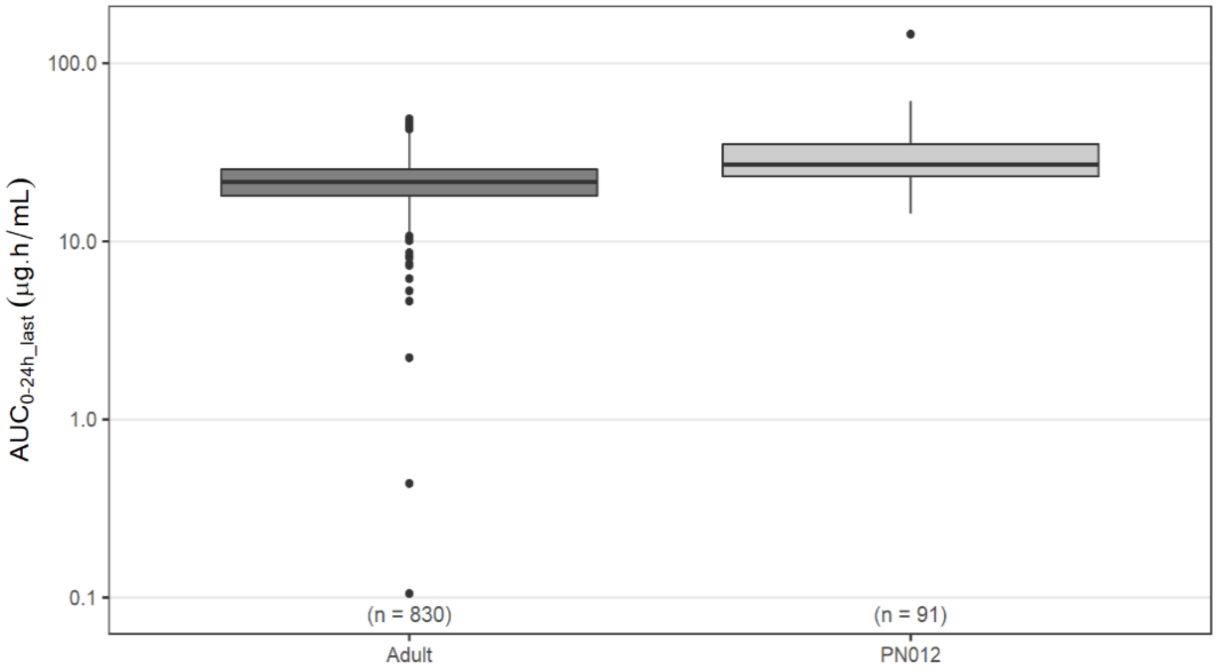

Supplement: Supplemental file 1 — Supplemental material. Download aac.00895-21-s0001.pdf, PDF file, 1.3 MB [file aac.00895-21-s0001.pdf]
